# The function of miRNAs in the immune system's inflammatory reaction to heart failure

**DOI:** 10.3389/fcvm.2024.1506836

**Published:** 2024-12-02

**Authors:** Kadierya Yibulayin, Maidinaimu Abulizi

**Affiliations:** Graduate School of Xinjiang Medical University, Urumqi, China

**Keywords:** miRNA, inflammation, TLR4, NF-κB, heart failure

## Abstract

Heart failure is the end stage of cardiovascular disease, with high morbidity and mortality rates worldwide. Heart failure is associated with long-term and insufficient inhibition of inflammatory response. miRNA is a class of endogenous, non-coding, single-stranded small RNA molecules, that can regulate gene expression through translational inhibition or degradation of targeted mRNA, widely regulate myocardial remodeling, inflammatory response, and other pathological processes, and play an important regulatory role in the occurrence and development of cardiovascular diseases. This article reviews the role of miRNA in the inflammatory response in heart failure.

## 1 Introduction

Heart failure is the end-stage of cardiovascular disease and has high morbidity and mortality rates globally (1). Despite the current treatment of heart failure including beta blockers, aldosterone receptor antagonists, cardiac enhancers, diuretics, and more, readmission and mortality rates are still high, placing a huge medical burden on society, and the search for new treatment strategies to prevent and delay heart failure is urgent (2). Recovery from myocardial injury requires the involvement of inflammatory cells, which play a key role in clearing damaged and necrotic cells, particularly macrophages, which play a key immunomodulatory role. Mild inflammation promotes tissue recovery, but chronic inflammation can lead to heart muscle dysfunction and poor ventricular remodeling. More recently, with a growing number of experimental and clinical studies linking inflammatory immune responses to the pathogenesis of heart failure, it has been recognized that heart failure is associated with chronic and poorly suppressed inflammatory responses, which are critical in the development of heart failure and can lead to molecular, cellular, and functional heart changes. During acute myocardial infarction (MI), the initial inflammation caused by plaque rupture triggers later reactions, potentially leading to more prolonged cardiac harm. Variations exist in the extent of inflammation recuperation post-reperfusion treatment, offering predictive insights into the emergence of ischemic heart failure. During extended periods of intense inflammatory reactions, the myocardium undergoes detrimental restructuring, culminating in heart failure (3). Safe, low-cost anti-inflammatory therapies need to be developed for early treatment and suppression of inflammatory responses to slow the progression of heart failure and deterioration of heart function. MiRNAs are a class of evolutionarily conserved non-coding single-stranded small molecule RNAs that contribute to the development of cardiovascular disease by promoting the degradation or translation inhibition of target mRNAs, regulating gene expression at the post-transcriptional level, and extensively regulating immune inflammatory responses, oxidative stress, cell proliferation, and apoptosis (4). Numerous scientific investigations have revealed the correlation between inflammatory markers and the severity and prognosis of chronic heart failure, suggesting that inflammation may be both a triggering factor and a consequence of heart failure. Although inflammation plays a critical role in the formation and development of diseases, the specific pathogenic mechanisms involved in the disease progression remain unclear.LI et al. indicated that there is a negative correlation between miR-21 and inflammatory factors in the peripheral blood of patients with chronic heart failure (CHF), suggesting that miR-21 may play a role in regulating inflammation within the pathogenesis of CHF (5). The role of miRNAs in regulating macrophages, endothelial cells, and inflammatory pathways in the inflammatory response to heart failure is reviewed.

## 2 miRNAs biology

MiRNAs are a class of evolutionarily conserved non-coding single-stranded small molecule RNAs of 19–25 nucleotides that regulate gene expression at the post-transcriptional level by binding to the mRNA's 3 ‘-non-translational domain (UTR) to promote mRNA degradation or translation inhibition (6). It is estimated that more than 60% of human protein-coding genes are targeted by miRNAs (7). miRNAs are first transcribed by RNA polymerase II (DGCR8) in the nucleus to produce primary miRNAs (8). Primary miRNAs contain stem-ring structures, each consisting of about 70 nucleotides, which are cleaved in the nucleus by microprocessor complexes consisting of Drosha III and DGCR8 to form 60–100 nucleotide precursor miRNAs (9). PremiRNAs transferred from the nucleus to the cytoplasm via Exportin 5 were broken down by Dicer III nucleic acid endocytose (RNA) into small double-stranded RNAs containing mature miRNAs, one of which binds to the Argonaute (AGO) protein and enters the RNA-induced silencing complex (RISC), while the other is degraded (10). Finally, mature miRNAs bind to target mRNAs, promote mRNA degradation or translational inhibition, and regulate gene expression at the post-transcriptional level. MiRNAs regulate various biological processes such as apoptosis, migration, proliferation, differentiation, inflammatory response, immune response, etc. Because of the wide range of target interactions, it also plays an important role in the development of many diseases, including cardiovascular disease (11).

## 3 Heart failure and inflammation

Inflammation is an important biological process for maintaining homeostasis in the body. When tissue damage occurs, the body immediately activates the innate and adaptive immune systems, triggering a robust inflammatory response (12). During myocardial injury, pathogen-associated molecular patterns (PAMPs), endogenous substances released by dying or stressed cells [damage-associated molecular patterns (damage-associated molecular patterns, DAMPs)] (13) and microbiota-associated molecular patterns (MAMPCs) released by the gut microbiota activate the receptors pattern recognition receptors, PRRs (14) upregulation of the expression of pro-inflammatory cytokines and chemokines, coupled with a rapid influx of neutrophils and monocytes from circulation to heart muscle injury sites, activates the innate immune system and triggers inflammatory responses (15). These upregulated inflammatory factors provide a range of protective effects on the heart muscle, promoting short-term adaptive stress in the heart, known as physiological inflammation. If the damage to the heart muscle is resolved, the acute inflammatory response disappears, and the heart muscle tissue returns to its fundamental equilibrium. Conversely, if myocardial damage cannot be resolved, macrophages and mast cells resident in the heart recruit additional white blood cells and plasma proteins to dysfunctional tissue areas in an attempt to restore the dynamic balance of heart tissue, leading to a persistent inflammatory response called a Para-inflammatory response (16). Once damage to the heart muscle continues, the inflammatory response becomes dysfunctional, leading to chronic inflammation. Excessive inflammation, excessive intensity, or inadequate inhibition can lead to persistent tissue damage and inappropriate healing, promote poor ventricular remodeling, and deterioration of heart function (17), thereby contributing to the heart failure process. A study comparing the serum levels of IL-1β in normal patients and those with chronic heart failure classified into functional classes II, III, and IV found that both IL-1β and NLRC4 expression levels increased in the chronic heart failure group. This increase was positively correlated with the severity of heart function, indicating that inflammatory responses play an important role in the development of heart failure. It can be inferred that inhibiting the activation of the NLRC4 inflammasome, thereby reducing the secretion of IL-1β, may represent a new therapeutic target for addressing the inflammatory response in chronic heart failure. Research has also indicated that monocyte chemoattractant protein-1 (MCP-1) plays a role in mediating the migration of monocytes and macrophages to damaged myocardial tissue, where it can recruit inflammatory factors and affect the injured myocardium, thereby exacerbating the inflammatory response and contributing to the progression of heart failure. Therefore, controlling inflammatory processes at an early stage to avoid persistent inflammation and heart damage is key to improving outcomes for patients with heart failure.

## 4 miRNA regulates inflammatory response in heart failure

The immune response is a kind of self-protective behavior of the body. The proper immune response can clear the pathogen and help the body recover. If the response is excessive, damage will occur to the body. The inflammatory response is the result of a violent immune response. MiRNAs are involved in heart failure progression by regulating endothelial inflammatory response and macrophage phenotype transformation. Research has shown that miR-939-5p is an inflammation-related miRNA, with serum levels in patients with chronic heart failure significantly higher than those in normal control groups. It has also been found that long non-coding RNAs (lncRNAs) can act as miRNA sponges to regulate their expression and target genes (18). By directly binding to miR-939-5p, they control inflammation-induced cell apoptosis, providing a new approach for reversing pathological inflammation in the treatment of CHF. MiRNAs have stable, hypoimmunogenic properties as therapeutics that can overcome the major limitations of small molecule and protein-based drugs (19), promising new therapeutic directions for modulating immune-inflammatory responses and preventing heart failure.

### 4.1 Macrophages

Immune cells play a key role in inflammatory processes: large numbers of immune cells infiltrate inflammatory regions, releasing inflammatory factors and exacerbating the inflammatory response. Immune-suppressant factors released by immune cells in turn promote the regression of inflammatory responses. In summary, inflammatory factors involved in the inflammatory response are closely related to immune cells, therefore inflammatory responses accompany immune responses (20). Macrophages are important effectors and regulators of inflammatory cascades. As the main immune cell population in cardiac tissue, macrophages are immediately involved in pathogen clearance and adaptive immune response in the event of myocardial injury.M1 macrophages are mainly involved in pro-inflammatory response and can be activated by lipopolysaccharide (LPS) to secrete pro-inflammatory cytokines such as interleukin-6 (IL-6), interleukin-12 (IL-4), tumor necrosis factor -α (tumor necrosis factor -α, TNF-α), etc.M2 macrophages, on the other hand, are primarily involved in the anti-inflammatory response, secreting interleukin-10 (IL-10), transforming growth factor-β (TGF-β), and others to promote tissue repair (21). miRNAs may have the ability to regulate macrophage polarization to pro-inflammatory or anti-inflammatory phenotypes. Promoting macrophage M1/M2 polarization can reduce inflammatory responses and adverse ventricular remodeling and may be a potential therapeutic target for heart failure. Ge et al. found that miR-155-5p metastasized to macrophages in mouse models of myocardial ischemia and reperfusion and promoted pro-inflammatory macrophage M1 polarization by activating the JAK2/STAT1 pathway, exacerbating inflammatory responses (22). The inhibition of miR-155-5p to control macrophage polarization may be a promising approach. Li et al. found that miR-155 directly targets the calcium-regulated thermostable protein CARHSP1, inhibits its expression, and indirectly downregulates TNF-α, thereby attenuating the inflammatory response of macrophages and reducing chronic inflammation (23). Li et al. showed that miRNA-202-3p protects heart function by regulating cholesterol acyltransferases and cholesterol hydrolases, inhibiting macrophage foam cell formation (24). miR-146a has been shown to reduce the activation of the inflammatory response by targeting the NF-κB inflammatory signaling pathway, thereby inhibiting the polarization of M1 macrophages (25). You et al. found that miR-223-3p reduced inflammatory responses in mice by decreasing the expression of inflammatory cytokines (26). Hirotake et al. have shown that extracellular vesicles (ECVs) can target regulatory transcription factor activation by delivering miR-192 to macrophages, leading to reduced expression of pro-inflammatory cytokines and chemokines and a reduction in the inflammatory response (27). The RNase III nuclease Dicer plays a crucial role in the conversion of pre-miRNA into mature miRNA. Previous studies have shown that the loss of Dicer-dependent regulation in miRNA biology may impair T cell-driven immune responses and promote the development of atherosclerosis by increasing the inflammatory response and lipid accumulation in lesion macrophages. JP et al. confirmed that platelet miRNA responds to myocardial I/R injury in a Dicer-dependent manner by specifically knocking out Dicer in megakaryocytes and platelets in mouse models (28). This positively influences the inflammatory response and myocardial healing process after infarction, thereby accelerating ventricular remodeling. KLF13 is a cardiac transcription factor involved in heart development and is a downstream gene targeted by miR-125a-5p (29). It has been reported that the nuclear translocation of KLF13 promotes the upregulation of CC chemokine ligand-5 (CCL5), activates M1 macrophage polarization, and inhibits M2 polarization through activation mediated by the MAPK pathway involving CCR1 and CCR5. This suggests that the influence of miR-125a-5p on macrophage polarization and the subsequent myocardial protection may be at least partially mediated by KLF13. These findings suggest that upregulating or downregulating miRNA expression, regulating their expression in macrophages, blocking the release of pro-inflammatory cytokines by macrophages, inhibiting the polarization of pro-inflammatory macrophages, and reducing foam cell production may be novel therapeutic options for heart failure by modulating the activation state of macrophages.

### 4.2 Endothelial cells

Endothelial cells are involved in and regulate inflammation, and inflammatory activation of endothelial cells is a key link in the development of many cardiovascular diseases (30). After myocardial ischemia and hypoxia, vascular endothelial cells were damaged, the homeostasis of active substances was disturbed, the inflammatory reaction was activated, pro-inflammatory cytokines were secreted, and inflammatory cells such as white blood cells were chemokayed. Therefore, reducing inflammatory responses in endothelial cells may reduce myocardial tissue damage, and endothelial inflammatory regulation may be a target for heart failure. TNF α is one of the cytokines involved in the inflammatory response. It induces endothelial cell activation to produce vascular cell adhesion molecule-1 (VCAM-1), which leads to a large number of inflammatory cells converging toward endothelial cells, promoting apoptosis and exacerbating the inflammatory response. Previous studies have found that patients with heart failure have significantly elevated levels of IL-6 and TNF-α in their peripheral blood compared to healthy individuals, and these levels are positively correlated with the severity of the condition (31).miRNA plays a regulatory role in inflammatory responses in vascular endothelial cells. Overexpression of miR-126, for example, activates the SIRT1/NrF2 signaling pathway, leading to reduced expression of pro-inflammatory cytokines such as IL-6 and TNF-α, which inhibit oxidative stress and inflammatory responses in cells (32). miR-126 is highly expressed in vascular endothelial cells, VCAM-1 is a target gene of miR-126, and miR-126 suppresses inflammatory response by binding to the mRNA non-coding region of VCAM-1, inhibiting VCAM-1 expression and reducing white blood cell adhesion to endothelial cells (33). miR-210 was found to reduce inflammatory cell density and macrophage accumulation in ischemia-damaged myocardial tissue (34). MiRNAs also regulate inflammatory responses by modulating NLRP3 inflammasome activity. Studies have shown that miR-22 protects endothelial cells by inhibiting the activation of the NLRP3 inflammatory small-body pathway, thereby reducing the levels of pro-inflammatory cytokines (35). Long et al. found that NLRP3 is a direct target of miR-223-3p, and miR-223-3p targets NLRP3 to inhibit small inflammatory body activation and reduce inflammatory response damage in endothelial cells (36). Endothelial cells produce endothelin, and levels of endothelin-1 (ET-1) are elevated in heart failure. Wang et al. have shown in rat models that miR-1-3p overexpression downregulates ET-1 expression, corrects the endothelial imbalance, and improves chronic heart failure through the miR-1-3p/ET-1 pathway in endothelial cells (37). Li et al. found in mouse models that miR-221-3p, expressed primarily in vascular endothelial cells, targets hypoxia-inducible factor-1 (HIF-1) to inhibit angiogenesis and worsen the myocardial injury. Elevated miR-221-3p may also suggest patients are in advanced stages of heart failure and may provide some evidence for treatment of severe heart failure. However, the inhibition of miR-221-3p improves heart function and slows heart failure and it may be a novel and effective molecular target for heart failure treatment (38). Antunes et al. demonstrated that miR-155-5p can be loaded onto nucleoplasm polysaccharide nanoparticles (CPAMs), remains intact and bioactive, passes to endothelial cells, reduces inflammatory responses, and increases endogenous cell protection (39). lncRNA acts as a “molecular sponge” for small RNAs, rescuing messenger RNA from post-transcriptional repression by miRNAs and regulating various pathological processes in endothelial dysfunction (40). Additionally, lncRNA inhibits endothelial cell inflammation by serving as a molecular sponge for miRNAs. These findings suggest that endothelial cells are critical for tissue repair and the inflammatory response. Improving the inflammatory environment in endothelial cells through miRNA may be an effective treatment for heart failure, opening new prospects for treating the inflammatory response in heart failure.

## 5 miRNAs regulate inflammatory reaction-related pathways in heart failure

miRNAs inhibit the macrophage-mediated immune response by regulating the NF-kB pathway and downstream TLR signaling.

### 5.1 NF-κB pathway

The nuclear factor-kappa-B (NF-κB) pathway mediates the inflammatory response and is a key regulator of nearly all biological immune processes. Activation of NF-κB leads to the accumulation and chemotaxis of inflammatory cells, releasing multiple pro-inflammatory cytokines such as IL-1, IL-6, TNF-α, etc. NF-κB activation is also a key component of the inflammatory response in cardiovascular diseases, so inhibition of the NF-κB pathway to improve endothelial inflammatory response may help alleviate heart function and thus heart failure. Yan et al. found in mouse models that miR-129-5p in exosomes derived from mesenchymal stem cells alleviate heart failure by targeting tumor necrosis factor receptor-associated factor 3 (TRAF3) and NF-κB signaling to reduce inflammatory responses and oxidative stress (41). Pu et al. found in rat myocardial infarction models that the endothelial receptor 1 (LOX1) of oxidized low-density lipoprotein (LDL) enhances cell damage by activating the NF-κB p65/Caspase-9 pathway, while mesenchymal stem cell-derived exosomes improve heart failure in rats by inhibiting miR-30e targeting LOX1 (42). Lin et al. showed that overexpression of miR-155 led to apoptosis and ventricular hypertrophy in mice, and SIRT1 improved cardiac function in heart failure mice by targeting the NF-κB/p65 pathway to inhibit miR-155 expression (43). He et al. found in mouse models that upregulation of miR-146a-3p, which targets IL-1 receptor-associated kinases and ubiquitin-ligase, reduced inflammatory cytokine production, reduced myocardial damage and improved cardiac function by inhibiting the NF-κB signaling pathway (44). Thus, miR-146a-3p may be a promising therapeutic and preventive strategy to improve heart failure. MAPKs and NF-κB signaling pathways are both strongly pro-inflammatory and pro-apoptotic signaling pathways, and Li et al. found that inhibition of miR-21 in mouse models exerts myocardial protective function by targeting MAPK kinase phosphatase-6 (MKP6) inactivation and targeting NF-κB and MAPK pathways (45). Xie et al. found that overexpression of miR-146a reduced NF-κB activation and affected the TLR-4/NF-κB signaling pathway, thereby reducing the inflammatory response (46). This provides new insights and research directions for targeting this pathway in anti-heart failure therapy.

### 5.2 TLR4 pathway

Toll-like receptor 4 (TLR4) is essentially a transmembrane protein, a pattern recognition receptor with abundant leucine-rich repeat sequences, belonging to the Toll-like receptor family and can be regulated by multiple miRNAs. Myocardial damage causes DAMPs or PAMPs to interact with TLR4, triggering a signaling cascade (47). Activation of TLR4 provides short-term protection to the heart, but long-term TLR4 signaling is detrimental to the heart, causing an increase in pro-inflammatory cytokines and cellular adhesion molecules, leading to increased inflammatory cells and poor cardiac remodeling. Therefore, the inhibition of TLR4 may be a potential therapeutic target to modulate cardiac dysregulation and long-term activation of immune responses. Yan et al. suggested that miR-181b upregulation could target the TLR4/NF-κB pathway to inhibit immune inflammatory response (48). Wang et al. found in a mouse model that miR-451a, abundant in adipose tissue, inhibits the inflammatory factor TNF-α, significantly increases the anti-inflammatory factor IL-10 associated with the TLR4 pathway, and reduces the inflammatory response (49). Feng et al. found that TLR4 expression was reduced when miR-135a was overexpressed in mouse models, and miR-135a targeting inhibited TLR4 activity, reduced inflammatory responses, and protected myocardial function (50). Increased levels of miR-135a may be a therapeutic response to heart failure inflammation. Li et al. found that the upregulation of miR-219a expression in mouse models enhanced the cardiac protection of sevoflurane by targeting the TLR4/MyD88 pathway to reduce melanoma 2 (AIM2) expression, reduce the inflammatory response and apoptosis (51). Thus, miR-219a may be a novel target for enhancing drug protection of heart muscle, Furthermore, miR-129 may reduce autophagy and apoptosis of cardiomyocytes induced by ISO or H2O2 through the miRNA-129/ATG14/AKT signaling pathway, and siRNA-MEG3 downregulates cardiomyocyte apoptosis (52), suggesting potential therapeutic value in treating maladaptive cardiac remodeling and heart failure. High mobility group box-1 protein (HMGB1) is a key mediator of inflammation, and high expression often leads to excessive inflammation. Zhang and others have shown that the overexpression of miR-708 inhibits TLR4-NF-κB signaling by directly targeting HMGB1, relieving myocardial cell damage and inflammation (53). Gong et al. demonstrated that miR-20a effectively protects cardiomyocytes by targeting TLR4 and inhibiting p38 MAPK/JNK signaling, reducing inflammatory responses in mouse models of ischemia-reperfusion injury (54).MiR-214-3p was also found to target TLR4 and inhibit inflammatory response (55).MiRNAs regulate inflammatory signaling pathways and interact with immune-inflammatory responses. Understanding the specific mechanisms of miRNAs could lead to new therapeutic strategies for heart failure based on the regulation of inflammatory immune responses.

## 6 Conclusion

Heart failure is the end stage of cardiovascular disease and one of the health conditions that threaten human life. The survival rate is still low and the long-term prognosis is generally poor. Inflammation plays an important role in the development of heart failure, and its regulation holds great promise for the treatment of heart failure. MiRNAs regulate inflammatory immune cascades, target macrophage polarization, and endothelial cell damage to prevent the progression of heart failure, and directly regulate inflammatory signaling pathways to slow heart failure progression. MiRNA-based therapies can be classified into two types: miRNA mimics and miRNA inhibitors. The former is a double-stranded RNA molecule that mimics miRNA, while the latter is a single-stranded RNA nucleotide designed to interfere with miRNA. In an animal model of heart failure in pigs, Hinke et al. found that the miR-21 inhibitor antimiR-21 had the most significant effect on miR-21 manipulation and inflammatory response signaling, and miR-21 was significantly suppressed, leading to suppression of inflammatory response and mitogen-activated protein kinase signaling pathways, reduced inflammatory response, and improved heart function (56). Although current research provides evidence of changes in relevant miRNA levels and demonstrates their signaling pathways, the analogs or inhibitors used in the studies may not fully represent the physiological roles of miRNAs. Additionally, the results from *in vitro* cell experiments are not entirely accurate, and the crucial role in the pathogenesis has not been established. This suggests that simply increasing the level of a single miRNA in the advanced stages of heart failure may be insufficient to reverse the progression of the disease.

Our understanding of the roles of miRNAs in signaling pathways is growing rapidly. MiRNAs promise new strategies and directions for the effective treatment of heart failure and are an attractive therapeutic target. The good stability of miRNA makes it more specific and stable for detecting heart failure. MiRNA can serve as a biomarker for heart failure, useful for early prediction and diagnosis of the disease, as well as for monitoring disease progression and prognosis. However, a single miRNA can regulate multiple genes, and one gene can be regulated by multiple miRNAs. The regulation of miRNAs is a complex regulatory network, and much more research is needed on the specific mechanisms. There are still some limitations to miRNA-based clinical applications. Continuing research to address the uncertainties of miRNA transport functions and mechanisms of action is promising, and we believe that miRNAs will have great research value and promising applications, both as drug targets for disease treatment and as molecular markers for disease diagnosis and prognosis. There are currently no miRNA-based drugs on the market and efficient and safe drug delivery methods, so the mechanism of action and safety efficacy of miRNAs in diseases must be further investigated. As we deepen our understanding of miRNA, we should continuously advance the innovation and optimization of relevant technologies, enrich and expand the theoretical foundation, and explore new diagnostic and therapeutic methods based on miRNA. This aims to address various issues in the heart failure process from a fresh perspective.

## References

[1] Maddox TM Januzzi JL Jr. Allen LA Breathett K Butler J Davis LL 2021 Update to the 2017 ACC expert consensus decision pathway for optimization of heart failure treatment: answers to 10 pivotal issues about heart failure with reduced ejection fraction: a report of the American College of Cardiology solution set oversight committee. J Am Coll Cardiol. (2021) 77:772–810. 10.1016/j.jacc.2020.11.022 33446410

[2] Dyavanapalli J Hora AJ Escobar JB Schloen J Dwyer MK Rodriguez J Chemogenetic activation of intracardiac cholinergic neurons improves cardiac function in pressure overload-induced heart failure. Am J Physiol Heart Circ Physiol. (2020) 319:H3–h12. 10.1152/ajpheart.00150.2020 32412778 PMC7474442

[3] Halade GV Lee DH. Inflammation and resolution signaling in cardiac repair and heart failure. EBioMedicine. (2022) 79:103992. 10.1016/j.ebiom.2022.103992 35405389 PMC9014358

[4] Kalayinia S Arjmand F Maleki M Malakootian M Singh CP. MicroRNAs: roles in cardiovascular development and disease. Cardiovasc Pathol. (2021) 50:107296. 10.1016/j.carpath.2020.107296 33022373

[5] Li W Li Y Jiang F Liu H. Correlation between serum levels of microRNA-21 and inflammatory factors in patients with chronic heart failure. Medicine (Baltimore). (2022) 101:e30596. 10.1097/MD.0000000000030596 36197244 PMC9509079

[6] Kilikevicius A Meister G Corey DR. Reexamining assumptions about miRNA-guided gene silencing. Nucleic Acids Res. (2022) 50:617–34. 10.1093/nar/gkab1256 34967419 PMC8789053

[7] Hill M Tran N. Global miRNA to miRNA interactions: impacts for miR-21. Trends Cell Biol. (2021) 31:3–5. 10.1016/j.tcb.2020.10.005 33189493

[8] Prasad A Sharma N Prasad M. Noncoding but coding: pri-miRNA into the action. Trends Plant Sci. (2021) 26:204–6. 10.1016/j.tplants.2020.12.004 33353820

[9] Hill M Tran N. miRNA interplay: mechanisms and consequences in cancer. Dis Model Mech. (2021) 14(4):dmm047662. 10.1242/dmm.047662 33973623 PMC8077553

[10] Ferragut Cardoso AP Banerjee M Nail AN Lykoudi A States JC. miRNA dysregulation is an emerging modulator of genomic instability. Semin Cancer Biol. (2021) 76:120–31. 10.1016/j.semcancer.2021.05.004 33979676 PMC8576067

[11] Groot M Lee H. Sorting mechanisms for microRNAs into extracellular vesicles and their associated diseases. Cells. (2020) 9(4):1044. 10.3390/cells9041044 32331346 PMC7226101

[12] Di Virgilio F Sarti AC Coutinho-Silva R. Purinergic signaling, DAMPs, and inflammation. Am J Physiol Cell Physiol. (2020) 318:C832–c5. 10.1152/ajpcell.00053.2020 32159362

[13] Castillo EC Vázquez-Garza E Yee-Trejo D García-Rivas G Torre-Amione G. What is the role of the inflammation in the pathogenesis of heart failure? Curr Cardiol Rep. (2020) 22:139. 10.1007/s11886-020-01382-2 32910299 PMC7481763

[14] Soysal P Arik F Smith L Jackson SE Isik AT. Inflammation, frailty and cardiovascular disease. Adv Exp Med Biol. (2020) 1216:55–64. 10.1007/978-3-030-33330-0_7 31894547

[15] Dauerman HL Ibanez B. The edge of time in acute myocardial infarction. J Am Coll Cardiol. (2021) 77:1871–4. 10.1016/j.jacc.2021.03.003 33858623

[16] Medzhitov R. Origin and physiological roles of inflammation. Nature. (2008) 454:428–35. 10.1038/nature07201 18650913

[17] Chong SY Zharkova O Yatim S Wang X Lim XC Huang C Tissue factor cytoplasmic domain exacerbates post-infarct left ventricular remodeling via orchestrating cardiac inflammation and angiogenesis. Theranostics. (2021) 11:9243–61. 10.7150/thno.63354 34646369 PMC8490508

[18] Chen C Zong M Lu Y Guo Y Lv H Xie L Differentially expressed lnc-NOS2P3-miR-939-5p axis in chronic heart failure inhibits myocardial and endothelial cells apoptosis via iNOS/TNFα pathway. J Cell Mol Med. (2020) 24:11381–96. 10.1111/jcmm.15740 32844595 PMC7576245

[19] Damase TR Sukhovershin R Boada C Taraballi F Pettigrew RI Cooke JP. The limitless future of RNA therapeutics. Front Bioeng Biotechnol. (2021) 9:628137. 10.3389/fbioe.2021.628137 33816449 PMC8012680

[20] Shao Y Saredy J Yang WY Sun Y Lu Y Saaoud F Vascular endothelial cells and innate immunity. Arterioscler Thromb Vasc Biol. (2020) 40:e138–e52. 10.1161/ATVBAHA.120.314330 32459541 PMC7263359

[21] Silvestre-Roig C Braster Q Ortega-Gomez A Soehnlein O. Neutrophils as regulators of cardiovascular inflammation. Nat Rev Cardiol. (2020) 17:327–40. 10.1038/s41569-019-0326-7 31996800

[22] Ge X Meng Q Wei L Liu J Li M Liang X Myocardial ischemia-reperfusion induced cardiac extracellular vesicles harbour proinflammatory features and aggravate heart injury. J Extracell Vesicles. (2021) 10:e12072. 10.1002/jev2.12072 33664937 PMC7902529

[23] Li X Kong D Chen H Liu S Hu H Wu T miR-155 acts as an anti-inflammatory factor in atherosclerosis-associated foam cell formation by repressing calcium-regulated heat stable protein 1. Sci Rep. (2016) 6:21789. 10.1038/srep21789 26899994 PMC4761895

[24] Li L Wu F Xie Y Xu W Xiong G Xu Y MiR-202-3p inhibits foam cell formation and is associated with coronary heart disease risk in a Chinese population. Int Heart J. (2020) 61:153–9. 10.1536/ihj.19-033 31956131

[25] Olivieri F Prattichizzo F Giuliani A Matacchione G Rippo MR Sabbatinelli J miR-21 and miR-146a: the microRNAs of inflammaging and age-related diseases. Ageing Res Rev. (2021) 70:101374. 10.1016/j.arr.2021.101374 34082077

[26] You D Qiao Q Ono K Wei M Tan W Wang C miR-223-3p inhibits the progression of atherosclerosis via down-regulating the activation of MEK1/ERK1/2 in macrophages. Aging (Albany NY). (2022) 14:1865–78. 10.18632/aging.203908 35202001 PMC8908932

[27] Tsukamoto H Kouwaki T Oshiumi H. Aging-associated extracellular vesicles contain immune regulatory microRNAs alleviating hyperinflammatory state and immune dysfunction in the elderly. iScience. (2020) 23:101520. 10.1016/j.isci.2020.101520 32927264 PMC7495115

[28] Schütte JP Manke MC Hemmen K Münzer P Schörg BF Ramos GC Platelet-derived MicroRNAs regulate cardiac remodeling after myocardial ischemia. Circ Res. (2023) 132:e96–e113. 10.1161/CIRCRESAHA.122.322459 36891903

[29] Gao L Qiu F Cao H Li H Dai G Ma T Therapeutic delivery of microRNA-125a-5p oligonucleotides improves recovery from myocardial ischemia/reperfusion injury in mice and swine. Theranostics. (2023) 13:685–703. 10.7150/thno.73568 36632217 PMC9830430

[30] Janaszak-Jasiecka A Siekierzycka A Płoska A Dobrucki IT Kalinowski L. Endothelial dysfunction driven by hypoxia-the influence of oxygen deficiency on NO bioavailability. Biomolecules. (2021) 11(7):982. 10.3390/biom11070982 34356605 PMC8301841

[31] Eskandari V Amirzargar AA Mahmoudi MJ Rahnemoon Z Rahmani F Sadati S Gene expression and levels of IL-6 and TNFα in PBMCs correlate with severity and functional class in patients with chronic heart failure. Ir J Med Sci. (2018) 187:359–68. 10.1007/s11845-017-1680-2 28889349

[32] Li J Yang C Wang Y. Mir-126 overexpression attenuates oxygen-glucose deprivation/reperfusion injury by inhibiting oxidative stress and inflammatory response via the activation of SIRT1/Nrf2 signaling pathway in human umbilical vein endothelial cells. Mol Med Rep. (2020) 23(2):165. 10.3892/mmr.2020.11804 33355373 PMC7789090

[33] Liu B Liu L Cui S Qi Y Wang T. Expression and significance of microRNA-126 and VCAM-1 in placental tissues of women with early-onset preeclampsia. J Obstet Gynaecol Res. (2021) 47:2042–50. 10.1111/jog.14732 33694224 PMC8251619

[34] Zaccagnini G Greco S Longo M Maimone B Voellenkle C Fuschi P Hypoxia-induced miR-210 modulates the inflammatory response and fibrosis upon acute ischemia. Cell Death Dis. (2021) 12:435. 10.1038/s41419-021-03713-9 33934122 PMC8088433

[35] Chi K Geng X Liu C Zhang Y Cui J Cai G LncRNA-HOTAIR promotes endothelial cell pyroptosis by regulating the miR-22/NLRP3 axis in hyperuricaemia. J Cell Mol Med. (2021) 25:8504–21. 10.1111/jcmm.16812 34296520 PMC8419175

[36] Long FQ Kou CX Li K Wu J Wang QQ. MiR-223-3p inhibits rTp17-induced inflammasome activation and pyroptosis by targeting NLRP3. J Cell Mol Med. (2020) 24:14405–14. 10.1111/jcmm.16061 33145937 PMC7754033

[37] Wang S Guo X Long CL Li C Zhang YF Wang J SUR2B/Kir6.1 Channel openers correct endothelial dysfunction in chronic heart failure via the miR-1-3p/ET-1 pathway. Biomed Pharmacother. (2019) 110:431–9. 10.1016/j.biopha.2018.11.135 30530045

[38] Li Y Yan C Fan J Hou Z Han Y. MiR-221-3p targets hif-1α to inhibit angiogenesis in heart failure. Lab Invest. (2021) 101:104–15. 10.1038/s41374-020-0450-3 32873879

[39] Antunes JC Benarroch L Moraes FC Juenet M Gross MS Aubart M Core-Shell polymer-based nanoparticles deliver miR-155-5p to endothelial cells. Mol Ther Nucleic Acids. (2019) 17:210–22. 10.1016/j.omtn.2019.05.016 31265949 PMC6610682

[40] Jayasuriya R Ganesan K Xu B Ramkumar KM. Emerging role of long non-coding RNAs in endothelial dysfunction and their molecular mechanisms. Biomed Pharmacother. (2022) 145:112421. 10.1016/j.biopha.2021.112421 34798473

[41] Yan F Cui W Chen Z. Mesenchymal stem cell-derived exosome-loaded microRNA-129-5p inhibits TRAF3 expression to alleviate apoptosis and oxidative stress in heart failure. Cardiovasc Toxicol. (2022) 22:631–45. 10.1007/s12012-022-09743-9 35546649

[42] Pu L Kong X Li H He X. Exosomes released from mesenchymal stem cells overexpressing microRNA-30e ameliorate heart failure in rats with myocardial infarction. Am J Transl Res. (2021) 13:4007–25. 34149995 PMC8205657

[43] Lin B Zhao H Li L Zhang Z Jiang N Yang X Sirt1 improves heart failure through modulating the NF-κB p65/microRNA-155/BNDF signaling cascade. Aging (Albany NY). (2020) 13:14482–98. 10.18632/aging.103640 33206628 PMC8202895

[44] He L Wang Z Zhou R Xiong W Yang Y Song N Dexmedetomidine exerts cardioprotective effect through miR-146a-3p targeting IRAK1 and TRAF6 via inhibition of the NF-κB pathway. Biomed Pharmacother. (2021) 133:110993. 10.1016/j.biopha.2020.110993 33220608

[45] Li Y Fei L Wang J Niu Q. Inhibition of miR-217 protects against myocardial ischemia-reperfusion injury through inactivating NF-κB and MAPK pathways. Cardiovasc Eng Technol. (2020) 11:219–27. 10.1007/s13239-019-00452-z 31916040

[46] Xie J Zhang L Fan X Dong X Zhang Z Fan W. MicroRNA-146a improves sepsis-induced cardiomyopathy by regulating the TLR-4/NF-κB signaling pathway. Exp Ther Med. (2019) 18:779–85. 10.3892/etm.2019.7657 31281454 PMC6591494

[47] Wang L Yang JW Lin LT Huang J Wang XR Su XT Acupuncture attenuates inflammation in microglia of vascular dementia rats by inhibiting miR-93-mediated TLR4/MyD88/NF-κB signaling pathway. Oxid Med Cell Longev. (2020) 2020:8253904. 10.1155/2020/8253904 32850002 PMC7441436

[48] Yan G Chen L Wang H Wu S Li S Wang X. Baicalin inhibits LPS-induced inflammation in RAW264.7 cells through miR-181b/HMGB1/TRL4/NF-κB pathway. Am J Transl Res. (2021) 13:10127–41. 34650685 PMC8507057

[49] Wang X Pham A Kang L Walker SA Davidovich I Iannotta D Effects of adipose-derived biogenic nanoparticle-associated microRNA-451a on toll-like receptor 4-induced cytokines. Pharmaceutics. (2022) 14(1):16. 10.3390/pharmaceutics14010016 35056912 PMC8780819

[50] Feng H Xie B Zhang Z Yan J Cheng M Zhou Y. MiR-135a protects against myocardial injury by targeting TLR4. Chem Pharm Bull (Tokyo). (2021) 69:529–36. 10.1248/cpb.c20-01003 34078799

[51] Li Y Xing N Yuan J Yang J. Sevoflurane attenuates cardiomyocyte apoptosis by mediating the miR-219a/AIM2/TLR4/MyD88 axis in myocardial ischemia/reperfusion injury in mice. Cell Cycle. (2020) 19:1665–76. 10.1080/15384101.2020.1765512 32449438 PMC7469529

[52] Mi S Huang F Jiao M Qian Z Han M Miao Z Inhibition of MEG3 ameliorates cardiomyocyte apoptosis and autophagy by regulating the expression of miRNA-129-5p in a mouse model of heart failure. Redox Rep. (2023) 28:2224607. 10.1080/13510002.2023.2224607 37338021 PMC10286679

[53] Zhang S Wang Y Wang P Xuan J. miR-708 affords protective efficacy in anoxia/reoxygenation-stimulated cardiomyocytes by blocking the TLR4 signaling via targeting HMGB1. Mol Cell Probes. (2020) 54:101653. 10.1016/j.mcp.2020.101653 32866662

[54] Gong XY Zhang Y. Protective effect of miR-20a against hypoxia/reoxygenation treatment on cardiomyocytes cell viability and cell apoptosis by targeting TLR4 and inhibiting p38 MAPK/JNK signaling. In Vitro Cell Dev Biol Anim. (2019) 55:793–800. 10.1007/s11626-019-00399-4 31444671

[55] Wang Q Liu Y Wu Y Wen J Man C. Immune function of miR-214 and its application prospects as molecular marker. PeerJ. (2021) 9:e10924. 10.7717/peerj.10924 33628646 PMC7894119

[56] Hinkel R Ramanujam D Kaczmarek V Howe A Klett K Beck C AntimiR-21 prevents myocardial dysfunction in a pig model of ischemia/reperfusion injury. J Am Coll Cardiol. (2020) 75:1788–800. 10.1016/j.jacc.2020.02.041 32299591

