# Supplementary figures and images for: The function of miRNAs in the immune system's inflammatory reaction to heart failure
Source: Front Cardiovasc Med. 2024 Dec 2;11:1506836. doi: 10.3389/fcvm.2024.1506836 (PMC11646975; doi:10.3389/fcvm.2024.1506836)

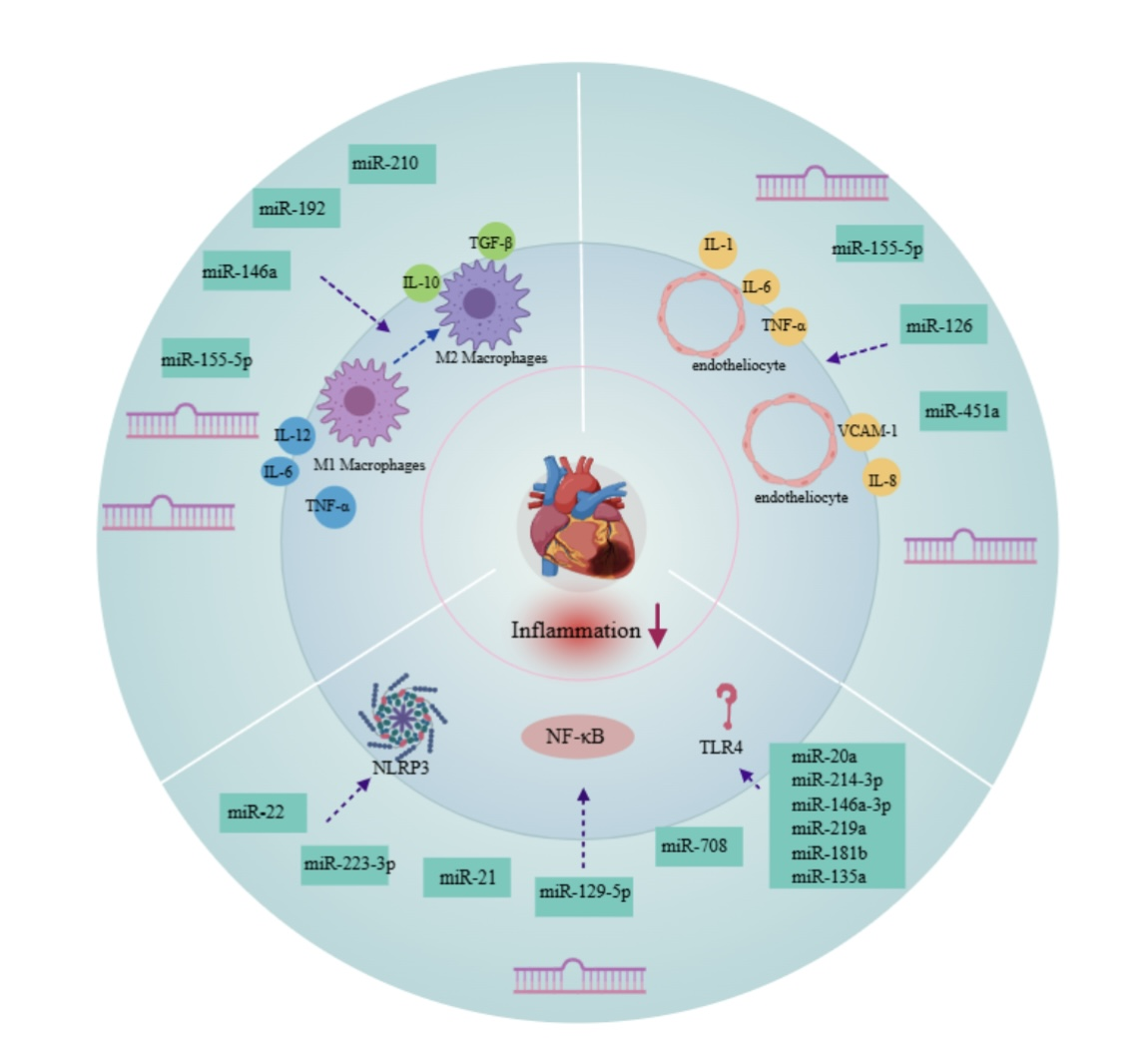

Supplement: Supplementary file 1 [file Image1.png]
